# Molecular duplexes featuring NH···N, CH···O and CH···π interactions in solid-state self-assembly of triazine-based compounds

**DOI:** 10.1098/rsos.220603

**Published:** 2022-11-02

**Authors:** Shazia Asghar, Shahid Hameed, Muhammad Nawaz Tahir, Muhammad Moazzam Naseer

**Affiliations:** ^1^ Department of Chemistry, Quaid-i-Azam University, Islamabad 45320, Pakistan; ^2^ Department of Physics, University of Sargodha, Sargodha 40100, Pakistan

**Keywords:** triazine derivatives, self-assembly, molecular duplexes, CH···O interactions, CH···π interactions, supramolecular synthons

## Abstract

Synthetic supramolecular structures constructed through the cooperative action of numerous non-covalent forces are highly desirable as models to unravel and understand the complexity of systems created in nature via self-assembly. Taking advantage of the low cost of 2,4,6-trichloro-1,3,5-triazine (cyanuric chloride) and the sequential nucleophilic substitution reactions with almost all types of nucleophiles, a series of six structurally related novel s-triazine derivatives **1–6** were synthesized and structurally characterized based on their physical, spectral and crystallographic data. The solid-state structures of all the six compounds showed intriguing and unique molecular duplexes featuring NH···N, CH···O and CH···π interactions. Careful analysis of different geometric parameters of the involved H-bonds indicates that they are linear, significant and are therefore responsible for guiding the three-dimensional structure of these compounds in the solid state. The prevalence of sextuple hydrogen bond array-driven molecular duplexes and the possibility of structural modifications on the s-triazine ring render these novel triazine derivatives **1–6** attractive as a platform to create heteroduplex constructs and their subsequent utility in the field of supramolecular chemistry and crystal engineering.

## 1. Introduction

Material properties are frequently dictated by the interactions and hence the way in which their constituent entities are assembled [1]. Consequently, molecular self-assembly has turned out to be an important 'bottom-up' approach to create intriguing materials of nano- and micro-structures, starting from simple components [2–6]. However, even with the best present-day synthetic approaches, accurate prediction of structures and the preparation of complementary nanostructures having efficiency equivalent to natural systems still remain a challenge and the ultimate goal of crystal engineers and supramolecular chemists [2–6]. The main reason is inadequate knowledge of intermolecular forces and poor understanding of the principles involved [7]. So far, numerous artificial self-assembly systems have been developed, employing a variety of supramolecular forces [8–11]. The most interesting are the systems involving arrays of multiple parallel or near-parallel H-bonds [12–18], developed primarily mimicking the DNA duplex having highly predictable intermolecular interactions and programmable sequence specificity.

Importance of weak hydrogen bonds such as CH···O and CH···π has recently been recognized in both natural and synthetic systems [19–24]. Although C–H groups are generally much weaker H-bond donors than hydrogens bound to heteroatoms, the CH···O/π interactions have been found to play important roles in physical, chemical and biological properties of a variety of substances [19–24]. Interestingly, the CH···O interactions are reported to constitute 20–25% of the total number of hydrogen bonds in proteins, playing key role in stabilizing the structures of proteins [25–30]. Similarly, the CH···π interactions have been found to be robust enough to stabilize a particular conformation of molecules for their higher order self-assembly [19,20]. These are documented as the main supramolecular forces in protein folding that stabilizes their secondary and tertiary structures [31,32], in addition to their role in arranging alkyl chains towards the phenyl group of amino acid residues and binding of proteins with cofactors and carbohydrates [33]. Owing to the recognition of their importance in natural systems, the CH···O/π interactions are now considered as vital supramolecular forces in various synthetic self-assembly processes, molecular and anion recognition events, and in crystal engineering field [34–38].

**Figure 1.**
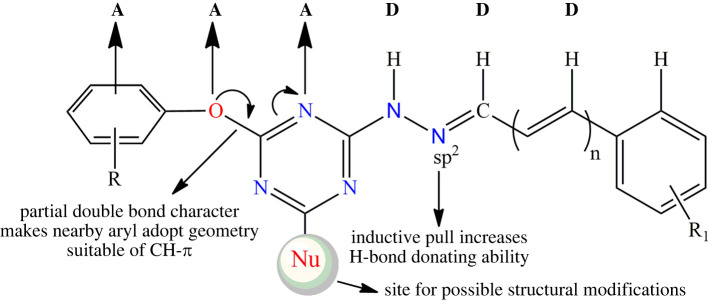
Proposed general structural features of synthesized triazine derivatives. Note: in cases where *n* = 0, neighbouring aryl CH may act as weak H-bond donor.

To date, a variety of self-assembled duplex systems involving arrays of conventional H-bonds have been recognized and subsequently used in the field of supramolecular chemistry [12–18]. However, the similar duplex systems involving weak hydrogen bonds such as CH···O/N/π are rarely identified and explored [39]. Keeping in view the importance of weak hydrogen bonds such as CH···O/N/π (vide supra), the duplex systems involving CH···O/N/π interactions are extremely important to be identified to meet the nature's selectivity in artificial systems. Stimulated by these observations and as continuation of our research interests in non-covalent interactions [40–46], herein we report the synthesis and structures of six novel triazine derivatives having AAADDD type H-bond acceptors and donor sites (π, O and N acceptors, and CH, CH and NH donors, respectively). Owing to the special structural features (figure 1), the formation of unique molecular duplexes featuring NH···N, CH···O and CH···π has been observed in the solid-state self-assembly of all the synthesized compounds.

## 2. Results and discussion

**Scheme 1.**
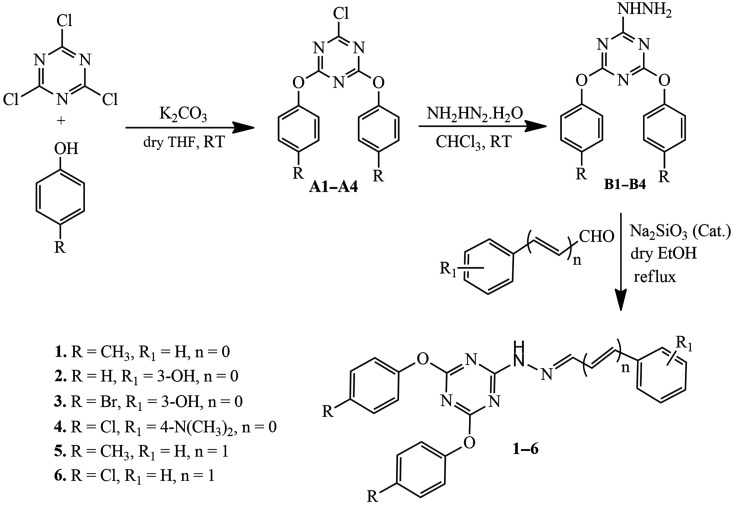
Synthesis of s-triazine derivatives **1–6.**

The s-triazine derivatives **1–6** were synthesized in three steps starting from readily available and inexpensive cyanuric chloride (scheme 1). In the first step, cyanuric chloride was reacted with a variety of phenols in dry tetrahydrofuran containing anhydrous potassium carbonate to obtain 2-chloro-4,6-diaryloxy-1,3,5-triazines **A** which were then treated with hydrazine monohydrate in chloroform solvent at room temperature in the second step to afford 2-hydrazinyl-4,6-diaryloxy-1,3,5-triazines **B**. Finally, the intermediates **B** were reacted with different aromatic aldehydes in refluxing dry ethanol containing the catalytic amounts of sodium hydrogen sulphite to furnish s-triazine derivatives **1–6** in good to excellent yields (scheme 1). All the synthesized triazine derivatives were at first characterized by their physical and spectral (FT-IR, ^1^H and ^13^C-NMR) data, and later by their single crystal X-ray diffraction analysis. The single crystals suitable for X-ray diffraction analysis of all the derivatives (table 1) were obtained by slow evaporation of their solution in ethanol solvent.

**Table 1.** X-ray crystallographic data of **1–6**.

crystal data	1	2	3	4	5	6
CCDC	2 155 046	2 155 047	2 155 048	2 155 049	2 155 050	2 155 051
chemical formula	C_24_H_21_N_5_O_2_	C_22_H_17_N_5_O_3_	C_22_H_15_Br_2_N_5_O_3_	C_24_H_20_Cl_2_N_6_O_2_	C_26_H_23_N_5_O_2_	C_24_H_17_Cl_2_N_5_O_2_
*M*_r_	411.46	399.40	557.21	495.36	437.49	478.32
crystal system, space group	monoclinic, *P*2_1_/*c*	triclinic, *P̄*1	monoclinic, *P*2_1_	triclinic, *P̄*1	triclinic, *P̄*1	monoclinic, *P*2_1_/*c*
temperature (K)	296	296	296	296	296	296
*a*, *b*, *c* (Å)	11.585 (2), 8.1214 (11), 22.565 (3)	11.9832 (12), 12.5490 (11), 13.8420 (11)	9.4582 (16), 22.182 (4), 10.407 (2)	11.8120 (11), 14.0117 (14), 16.0983 (14)	12.9628 (11), 13.0656 (12), 15.2100 (13)	16.525 (9), 8.180 (7), 18.726 (13)
α, β, γ (°)	90.391 (10)	106.918 (4), 93.462 (5), 99.538 (5)	90.648 (13)	99.163 (3), 108.034 (4), 104.982 (6)	68.900 (4), 79.785 (4), 77.004 (4)	109.48 (3)
*V* (Å^3^)	2123.1 (6)	1950.9 (3)	2183.3 (7)	2362.9 (4)	2328.6 (4)	2386 (3)
*Z*	4	4	4	4	4	4
radiation type	Mo *K*α	Mo *K*α	Mo *K*α	Mo *K*α	Mo *K*α	Mo *K*α
µ (mm^−1^)	0.09	0.09	3.75	0.31	0.08	0.30
crystal size (mm)	0.44 × 0.22 × 0.16	0.37 × 0.33 × 0.22	0.38 × 0.26 × 0.22	0.38 × 0.32 × 0.24	0.44 × 0.35 × 0.28	0.38 × 0.34 × 0.28
data collection						
diffractometer	Bruker Kappa APEXII CCD	Bruker Kappa APEXII CCD	Bruker Kappa APEXII CCD	Bruker Kappa APEXII CCD	Bruker Kappa APEXII CCD	Bruker Kappa APEXII CCD
absorption correction	multi-scan (*SADABS*; Bruker, 2005)	multi-scan (*SADABS*; Bruker, 2005)	multi-scan (*SADABS*; Bruker, 2005)	multi-scan (*SADABS*; Bruker, 2005)	multi-scan (*SADABS*; Bruker, 2005)	multi-scan (*SADABS*; Bruker, 2005)
*T*_min_, *T*_max_	0.675, 0.746	0.675, 0.746	0.345, 0.550	0.675, 0.746	0.675, 0.746	0.675, 0.746
no. of measured, independent and observed [*I* > 2σ(*I*)] reflections	13 303, 4617, 2420	22 828, 7656, 3849	12 636, 7647, 3970	26 688, 8743, 4532	25 415, 8613, 4140	14 629, 5204, 2763
*R*_int_	0.056	0.073	0.058	0.071	0.062	0.058
(sin θ/λ)_max_ (Å^−1^)	0.639	0.617	0.606	0.606	0.606	0.639
refinement						
*R*[*F*^2^ > 2σ(*F*^2^)], *wR*(*F*^2^), *S*	0.056, 0.151, 1.00	0.054, 0.146, 0.96	0.062, 0.150, 0.96	0.058, 0.153, 0.98	0.057, 0.169, 0.98	0.056, 0.178, 1.01
no. of reflections	4617	7656	7647	8743	8613	5204
no. of parameters	282	543	521	617	600	298
no. of restraints	—	—	1	—	—	—
H-atom treatment	H-atom parameters constrained	H-atom parameters constrained	H atoms treated by a mixture of independent and constrained refinement	H-atom parameters constrained	H-atom parameters constrained	H-atom parameters constrained
Δ>max, Δ>min (e Å^−3^)	0.19, −0.20	0.15, −0.18	0.96, −0.53	0.28, −0.32	0.24, −0.22	0.46, −0.41

**Figure 2.**
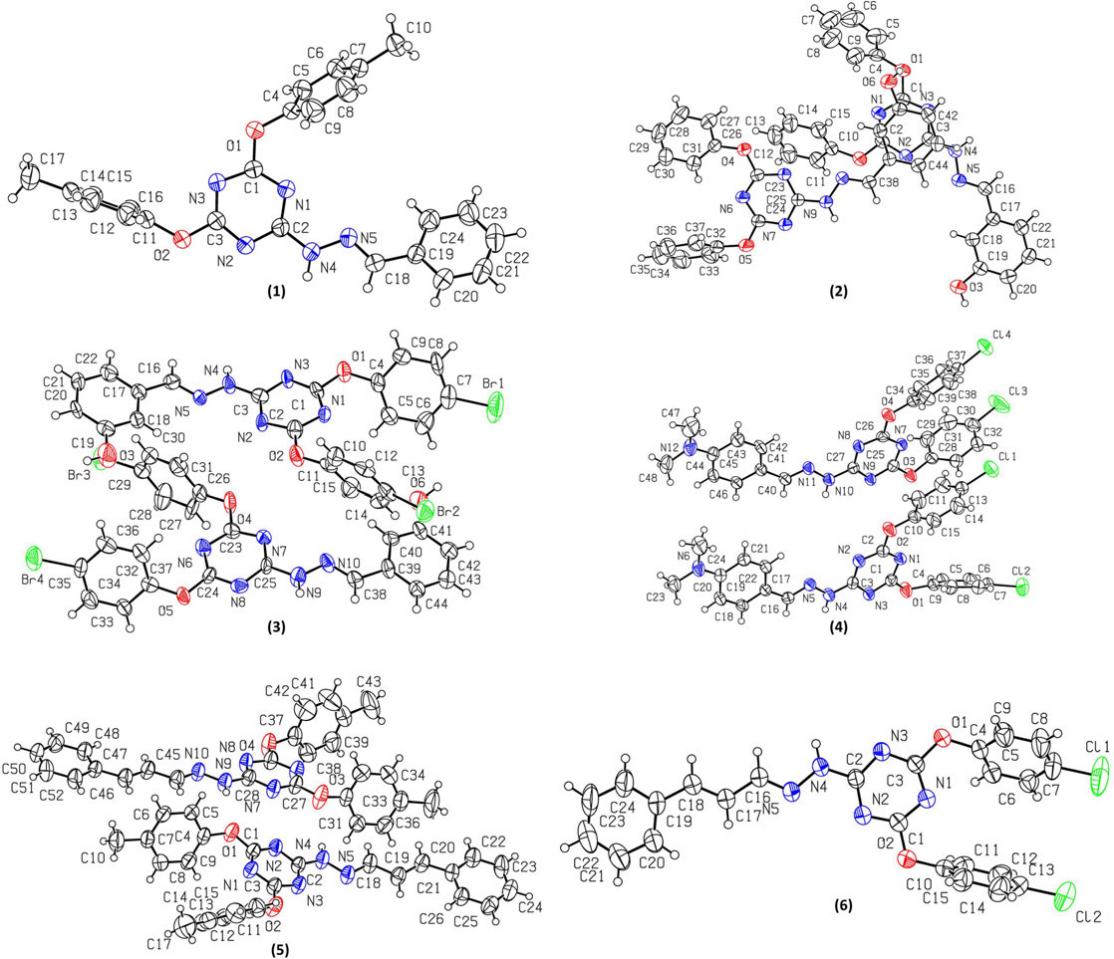
The molecular structures (Oak Ridge thermal ellipsoid plot (ORTEP) diagram) of triazine derivatives **1–6**. Displacement ellipsoids are drawn at 50% probability level.

The molecular structures of triazine derivatives **1–6** along with crystallographic numbering schemes are illustrated in figure 2. Interestingly, the compounds **1, 3** and **6** crystallize in monoclinic system with space group *P*2_1_/*c_,_ P*2_1_ and *P*2_1_/*c*, respectively, whereas compounds **2**, **4** and **5** crystallize in triclinic system, all having *P̄*1 space group. Despite having almost the same molecular core, the structures of **1–6** are not isomorphous, reflecting the impact of the substituents on the molecular packing (table 1). The compounds **1** and **6** have one molecule in the asymmetric unit whereas, two different/independent molecules/conformers having slightly different bond lengths, dihedral angles and torsions angles were observed in the unit cell of compounds **2–5** (figure 2). Selected geometric parameters for all the six compounds are presented in the electronic supplementary material, table S1. The magnitude of the standard uncertainty values linked with these geometric parameters prohibits description of any definitive trends across the series. The central core comprising the imino group of hydrazone and triazinyloxy moiety is essentially planar in the crystal structures of all six compounds. This planarity can be attributed to the partial double bond character of both C-N and C-O bonds involving triazine ring due to significant delocalization of nitrogen and oxygen lone pairs towards the triazine ring. This is clearly indicated by the shorter bond lengths of C-N and C-O bonds [N4-C2 1.347(2) Å and O2-C3 1.342(2) Å in **1**, N4-C3 1.341(3) Å, N9-C25 1.339(3) Å and O1-C1 1.355(3) Å, O5-C24 1.344(3) Å in **2,** N4-C3 1.331(16) Å, N9-C25 1.348(15) Å and O1-C1 1.364(13) Å, O5-C24 1.343(15) Å in **3,** N4-C3 1.336(4) Å, N9-C25 1.344(3)Å and O1-C1 1.357(3) Å, O3-C25 1.360(3) Å in **4,** N4-C2 1.340(3) Å, N9-C28 1.340(3) Å and O1-C1 1.348(3) Å, O3-C27 1.348(3) Å in **5,** N4-C2 1.352(3) Å and O1-C3 1.360(3) Å in **6**] (electronic supplementary material, table S1). The aryl ring attached to the oxygen of central/planar core is tilted/twisted and in some cases present nearly at right angle compared with the triazine plane [C3-O2-C11-C12 93.7(3)° in **1**, C1-O1-C4-C5 −121.9(3)° and C24-O5-C32-C33 123.5(3)° in **2**, C1-O1-C4-C5 46.9(13)° and C24-O5-C32-C33 141.3(10)° in **3**, C1-O1-C4-C5 −143.8(3)° and C25-O3-C28-C29 −87.1(4)° in **4**, C1-O1-C4-C5 132.4(2)° and C27-O3-C30-C31 119.4(3)° in **5**, C3-O1-C4-C9 −123.6(3)° in **6**]. The deviation of aryl ring plane from the right-angled geometry can be attributed to the varying degree of conjugation of oxygen with neighbouring (hetero)aromatics (figure 1). By contrast, the other aryl ring attached to the central/planar core in all the compounds except **1** is almost present in the same plane as triazine ring owing to the extended conjugation [N5-C18-C19-C20 165.5(2)° in **1**, N5-C16-C17-C22 174.3(3)°, N10-C38-C39-C44 173.0(3)° in **2**, N5-C16-C17-C22 176.4(10)°, N10-C38-C39-C44 −173.7(14)° in **3**, N5-C16-C17-C22 2.3(5)°, N11-C40-C41-C42 1.9(5)° in **4**, C19-C20-C21-C22 170.3(3)°, C45-C46-C47-C52 176.5(3)° in **5** and C17-C18-C19-C24 176.6(3)° in **6**]. The slightly more tilt observed in the case of compound **1** may be attributed to the packing effect. This particular arrangement of central core and the two attached aryl rings offers three H-bond donors [NH, CH (imine) and CH (aryl/sp^2^)] and three H-bond acceptors [N (triazine ring), O (bridged between (hetero)aromatic rings, π system (aryl)]. Interestingly, the presence of sp^2^-hybridized nitrogen atom in the central core increases acidity of both nearby NH and CH groups, making them better H-bond donors. As the strength of any hydrogen bond is more dependent on donor acidity than acceptor basicity [22], an expected consequence of this particular arrangement and increased acidity of NH and CH groups is the facile formation of molecular duplexes featuring NH···N, CH···O and CH···π interactions (vide infra).

**Figure 3.**
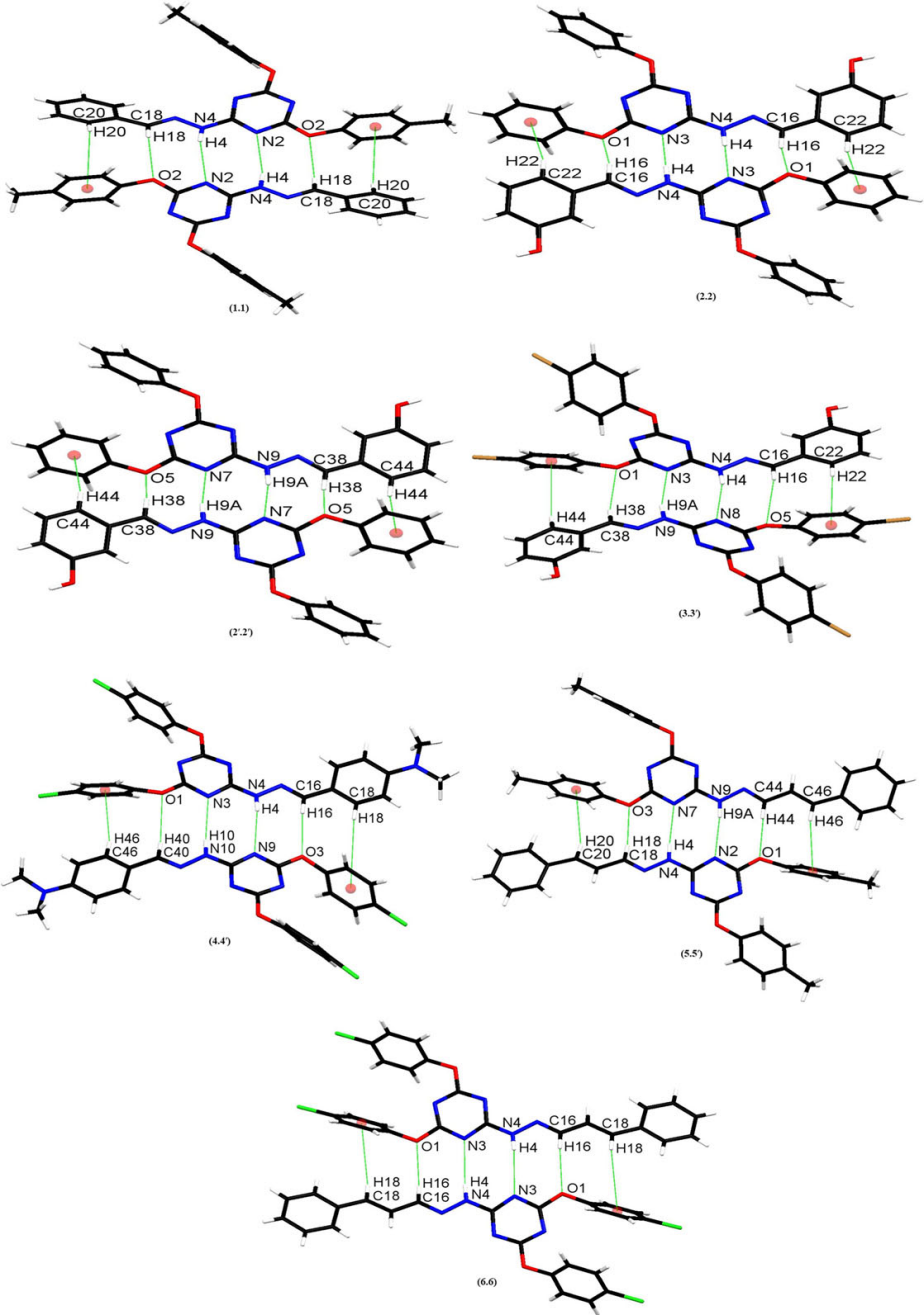
Molecular duplexes observed in the solid-state structures of triazine derivatives **1–6.**

As shown in figure 3, all the six solid-state structures feature a molecular duplex driven by NH···N, CH···O and CH···π interactions, and this clearly is the predominant supramolecular synthon operating in each of the crystal structures; geometric parameters associated with this synthon are listed in table 2. In addition to observation of a centrosymmetric molecular duplex in the solid-state structure of compounds **1** (1.1) and **6** (6.6) that has one molecule in their asymmetric units, a centrosymmetric molecular duplex has also been observed for compound **2** having two independent molecules in the crystal structure. In this compound, two different centrosymmetric duplexes [(2.2) and (2′.2′)] have been observed. However, all other compounds of the series i.e. **3**, **4** and **5** having two different conformers/independent molecules in their unit cells showed the formation of duplexes between two different conformers [duplexes of type (3.3′), (4.4′) and (5.5′), respectively] (figure 3).

**Table 2.** Geometric parameters associated with the hydrogen bonds of molecular duplexes in **1–6**.

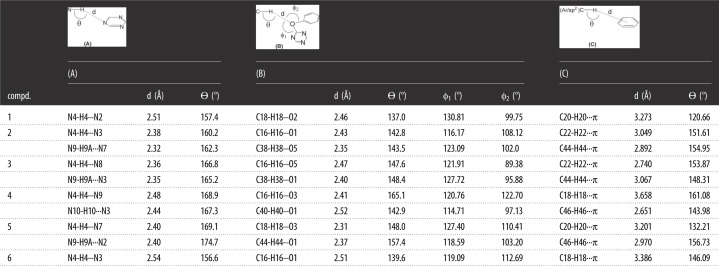

Over and beyond the NH···N, CH···O and CH···π interactions involved in the formation of molecular duplexes, the crystal packing of **1** (*R* = 4-CH_3_, *R*_1_ = H, *n* = 0) is dominated by π···π, CH···π and CH···N interactions. The self-complementary molecular duplex of **1** interacts with a neighbouring duplex by means of an antiparallel π···π [C5⋯C6 3.337 Å] stacking and a CH···π [C24-H24···C6 2.828 Å] interaction, resulting in the formation of one-dimensional tapes (electronic supplementary material, figure S1). These tapes then connect to the neighbouring tapes by means of C-H···π [C12-H12···C14 2.769 Å] and CH···N [C9-H9···N2 2.692 Å] interactions, making an overall three-dimensional network structure (electronic supplementary material, figure S2). Similar one-dimensional tapes are observed in compound **2** (*R* = H, *R*_1_ = 3-OH, *n* = 0) and **3** (*R* = 4-Br, *R*_1_ = 3-OH, *n* = 0), both having a strong H-bonding OH group. Owing to the presence of two different/independent molecules/conformers in the unit cell of **2**, two self-complementary molecular duplexes, (2.2) and (2′.2′), interact with neighbouring duplexes by means of OH···N [O3-H3···N2 2.221 Å and O6-H6A···N8 2.137 Å] and CH···O [C18-H18···O3 2.399 Å, C20-H20···O2 2.458 Å and C40-H40···O6 2.473 Å, C42-H42···O4 2.448 Å] contacts. These tapes of **2** joins with the neighbouring tapes with the help of π···π [C2···C30 3.363 Å] and CH···π [C33-H33···C13 2.891 Å] interactions, making an overall three-dimensional network structure of **2** (electronic supplementary material, figure S2). In contrast with the duplexes of **2** and as pointed out above, a molecular duplex of **3** featuring two independent conformers (3.3′) is formed despite having two different/independent molecules/conformers in its unit cell. These duplexes interact with each other by means of a bifurcated OH···N and OH···O [O3-H3···N7 2.218 Å and O3-H3···O4 2.603 Å] and two CH···O [C20-H20···O4 2.491 Å, C40-H40···O3 2.489 Å] interactions forming one-dimensional tapes (electronic supplementary material, figure S1). These tapes connect with the neighbouring tapes by CH···π [C6-H6···O6 2.648 Å], CH···Br [C33-H33···Br 2.878 Å] and a lone pair···π [Br4···C7 3.518 Å] interactions to extend a three-dimensional network structure of **3** (electronic supplementary material, figure S2). Interestingly, the tape structures observed for **1–3** are not observed for compound **4**. In compound **4,** each molecular duplex featuring two independent conformers (3.3′) stack on neighbouring duplex by means of CH···N [C8-H8···N8 2.610 Å] and CH···π [C9-H9···C42 2.885 Å] interactions (electronic supplementary material, figure S1). These stacked duplexes attach to the neighbouring stacked duplexes with the help of CH···Cl [C19-H19···Cl3 2.903 Å], CH···O [C23-H23B···O1 2.651 Å] and a centrosymmetric lone pair···π [Cl2···C6 3.428 Å] interactions providing three-dimensional network structure of **4** (electronic supplementary material, figure S2). Supramolecular tapes featuring molecular duplexes (5.5′) are also formed for **5** by means of C-H···N [C49-H49···N5 2.675 Å] and C-H···O [C50-H50···O2 2.598 Å] interactions (electronic supplementary material, figure S1). These tapes are interconnected by means of C-H···π [C41-H41···C49 2.878 Å] and CH···N [C34-H34···N2 2.699 Å] interactions in the three-dimensional network structure of **5** (electronic supplementary material, figure S1). The self-complementary molecular duplexes observed for **6**, however, form one-dimensional zigzag chains by means of CH···N [C6-H6···N5 2.806 Å], a bifurcated CH···N/O [C5-H5···N2 2.836 Å and C5-H5···O2 2.560 Å] and C-H···O [C11-H11···O2 2.785 Å] interactions (electronic supplementary material, figure S1). These chains connect to the neighbouring chains by C-H···π [C21-H21···C24 2.991 Å, C15-H15···C5 2.804 Å] and CH···Cl [C22-H22···Cl1 3.010 Å] interactions, furnishing three-dimensional network structure of **6** (electronic supplementary material, figure S2).

Directionality is considered as one of the most important features of a hydrogen bond to distinguish it from the mere London dispersion forces. Generally it is believed that the linear bonds (150° < *ϴ* < 180°) are structurally more significant due to the dipole–monopole and dipole–dipole contribution to the electrostatic energy which is a maximum at *ϴ* = 180° and zero at *ϴ* = 90° [22]. Careful analysis of the bond angles *ϴ* in table 2 demonstrates that the NH···N hydrogen bonds involved in the formation of molecular duplexes are relatively linear and the most significant. However, the same angle *ϴ* associated with C-H···O and C-H···π interactions involved in the formation of molecular duplexes is slightly more bent as compared with *ϴ* associated with NH···N hydrogen bonds, although it is still in the range of significant interactions. Interestingly, most of the CH···O distances are comparable to NH···N distances (please see d (Å) in table 2). As compared with the distances of NH···N and CH···O, the distances of CH···π [CH (aryl/sp^2^) to centre of aryl ring of neighbouring molecule] interactions are slightly longer, although falls within the limits [47]. However, the angle associated with these interactions clearly indicates the significant role of these interactions, as many CH···π interactions are clustered around a distance of C–π centre approximately 4–6 Å and angle *ϴ* approximately 120°–150° [47]. It is important to mention here that most of the CH···π interactions having greater angle *ϴ* have shorter distances, although whole data prohibits description of any definitive trends across the series (See d (Å) under column C in table 2). The angle ϕ is usually measured to see O/N-atom lone pair directionality. The angles ϕ1 and ϕ2 in table 2 have been observed in the range of 114.71–130.81° and 89.38–122.70° indicating the sidewise approach of the H-bond donors [48]. The variation of angles ϕ in these compounds also indicates the formation of various conjugation systems of oxygen with the adjacent aromatic rings. It can finally be inferred from the data gathered in table 2 that the cooperativity of NH···N, CH···O and CH···π interactions observed in triazine derivatives **1–6** make the otherwise weak CH···O and CH···π interactions strong and significant. Owing to this cooperativity, the molecular duplexes are observed even in compounds having strong competing H-bonding interactions, especially those involving OH groups. Therefore, the observed molecular duplexes are robust and are responsible for guiding the three-dimensional structures of these compounds in the solid state.

## 3. Conclusion

In summary, we have synthesized and crystallographically characterized six new structurally similar s-triazine derivatives **1–6** in order to see the formation of molecular duplexes through the cooperative action of NH···N, CH···O and CH···π interactions. Fascinatingly, the solid-state structures of all the six compounds showed the formation of expected molecular duplexes. The analysis of different geometric parameters clearly indicates the linear and significant nature of the involved non-covalent interactions highlighting their cooperative and interdependent nature, mutually influencing the strength of each other. Owing to the cooperative role of NH···N, CH···O and CH···π interactions, the observed molecular duplexes are robust supramolecular synthons, responsible for guiding the three-dimensional structures of synthesized triazine derivatives. Exploring the detailed structural and energetic impact of this cooperativity and the formation of duplexes will be interesting in host-guest chemistry, crystal engineering and other fields of supramolecular chemistry, especially when there is a possibility of structural modifications on the s-triazine ring (figure 1) keeping all the observed AAADDD type H-bond acceptors and donor sites intact.

## 4. Experimental section

### 4.1. General procedure for the synthesis 2-hydrazinyl-4,6-substituted-diphenoxy-1,3,5-triazines (B1–B4)

The 2-chloro-4,6-diaryloxy-1,3,5-triazine (**A1–A4**) (0.0066 mol) was dissolved in 30 ml of chloroform and added dropwise to the solution of hydrazine monohydrate (2 ml, 0.326 mol) in chloroform with constant stirring over a period of 16 h at room temperature. The reaction mixture was further stirred for another 6–8 h. After the completion of reaction as indicated by TLC, the mixture was washed with 3 × 30 ml of water in order to remove excess of hydrazine hydrate. The chloroform layer was then collected and dried over anhydrous sodium sulfate. The solid obtained after evaporation of chloroform under vacuum was recrystallized from ethanol to get respective pure product (**B1–B4**).

#### 4.1.1. 2-Hydrazinyl-4,6-bis(4-tolyloxy)–1,3,5-triazine (B1)

**Yield**: 68%; **m.p.**: 185–187°C; **R*_f_***: 0.62 (chloroform : methanol; 9 : 1); **^1^H-NMR** (300 MHz, DMSO-d_6_): *δ* (ppm) 9.19 (s, 1H, NH), 7.22–7.19 (4H, m, H-6,6′,10,10′), 7.12–7.09 (2H, d, *J* = 8.4 Hz, H-5,9), 7.10 (2H, d, *J* = 8.4 Hz, H-5′,9′), 4.34 (2H, s, NH_2_), 2.31, 2.29 (6H, s, 2 × CH_3_); **^13^C-NMR** (75 MHz, DMSO-d_6_):*δ* **(**ppm) 172.5 (C-1), 171.6, 169.5 (C-2,3),150.1, 150.0 (C-4,8), 135.0, 134.9 (C-7,11), 130.3, 130.1 (C-6,6′,10,10′), 122.2, 121.8 (C-5,5′,9,9′), 20.8, 20.8 (2 × CH_3_)

#### 4.1.2. 2-Hydrazinyl-4,6-diphenoxy-1,3,5-triazine (B2)

**Yield**: 67%; **m.p.**: 106–108°C [Lit. 107] [49]; **R_*f*_**: 0.51 (chloroform : methanol; 9 : 1); **^1^H-NMR** (300 MHz, DMSO-d_6_): *δ* (ppm) 9.22 (s, 1H, NH), 7.44–7.36 (4H, m, H-6,6′,10,10′), 7.27–7.15 (6H, m, H-5,5′,9,9′,7,11), 4.34 (2H, d, *J* = 2.7 Hz, NH2), **^13^C-NMR** (75 M Hz, DMSO-d_6_): *δ* (ppm) 172.3 (C-1) 171.4, 169.6 (C-2,3), 152.2, 152.2 (C-4,8) 129.9, 129.8 (C-6,6′,10,10′), 125.9 (C-7,11) 122.1(C-5,5′,9,9).

#### 4.1.3. 2,4-bis(4-Bromophenoxy)-6-hydrazinyl-1,3,5-triazine (B3)

**Yield**: 66%; **m.p.**: 183–185°C; **R*_f_***: 0.45 (chloroform : methanol; 9 : 1); **^1^H-NMR** (300 MHz, DMSO-d_6_) (300 MHz, DMSO-d_6_): *δ* **(**ppm) 9.27 (s, 1H, NH), 7.61–7.55 (4H, m, H-6,6′,10,10′), 7.24–7.13 (4H, m, H-5,5′,9,9′), 4.37 (2H, s, NH_2_); **^13^C-NMR** (75 MHz, DMSO-d_6_): *δ* **(**ppm) 172.0 (C-1), 171.2, 169.4 (C-2,3), 151.5,151.4 (C-4,8), 132.7, 132.6 (C-6,6′,10,10′), 124.6, 124.5 (C-5,5′,9,9′), 118.2, 118.1 (C-7,11).

#### 4.1.4. 2,4-bis(4-Chlorophenoxy)-6-hydrazinyl-1,3,5-triazine (B4)

**Yield**: 60%; **m.p.**: 166–168°C; **R*_f_***: 0.49 (chloroform : methanol; 9 : 1); **^1^H-NMR** (300 MHz, DMSO-d_6_) (300 MHz, DMSO-d_6_): *δ* **(**ppm) 9.27 (s, 1H, NH), 7.47–7.43 (4H, m, H-6,6′,10,10′), 7.27 (2H, d, *J =* 9.0 Hz, H-5,9), 7.21 (2H, d, *J =* 9.0 Hz, H-5′,9′), 4.37 (2H, s, NH_2_); **^13^C-NMR** (75 M Hz, DMSO-d_6_): *δ* **(**ppm) 172.1 (C-1), 171.2, 169.4 (C-2,3), 151.0, 150.9 (C-4,8), 130.1, 130.0 (C-6,6′,10,10′), 129.8, 129.7 (C-7,11) 124.1, 124.1 (C-5,5′,9,9′).

### 4.2. General procedure for the synthesis 2-(substituted-benzylidenehydrazinyl)-4,6-substituted-diphenoxy-1,3,5-triazine (1–6)

The 2-hydrazinyl-4,6-diphenoxy-1,3,5-triazine **(B1–B4)** (0.01 mol) was added to a 20 ml of dry ethanol and the mixture was stirred for 15 min at room temperature, followed by the addition of 0.012 mol of substituted aldehydes and catalytic amount of sodium hydrogen sulphite. The whole reaction mixture was then refluxed for 6–12 h, which resulted in the appearance of a solid product. After completion of the reaction as indicated by TLC, the solid product was filtered, washed with cold ethanol and recrystallized from a mixture of acetonitrile and ethanol to afford respective pure product (**1–6**).

#### 4.2.1. 2-(2-Benzylidenehydrazinyl)-4,6-bis(*p*-tolyloxy)-1,3,5-triazine (1)

**Yield**: 70%; **m.p.**: 218–219°C; **R*_f_***: 0.70 (*n*-Hexane : ethyl acetate; 6 : 4); **IR** (ATR, ῡ): cm^−1^ 3243 (N-H stretch.), 3030 (C_sp2_—H stretch.), 2918 (C_sp3_—H stretch.), 1604 (C=N stretch.), 1570, 1504 (2 × C=C stretch.), 1359, 1199 (2 × C-O stretch.); **^1^H-NMR** (300 MHz, DMSO-d_6_): *δ* (ppm) 11.79 (1H, s, NH), 8.16 (1H, s, H-12), 7.62 (2H, dd, *J* = 7.5, 4.2 Hz, H-14,18), 7.43–7.40 (3H, m, H-15,16,17), 7.26–7.20 (4H, m, H-6,6′,10,10′), 6.71–7.08 (4H, m,H-5,5′,9,9′), 2.34, 2.31 (6H, s, 2 × CH_3)_; **^13^C-NMR** (75 MHz, DMSO-d_6_): *δ* (ppm) 172.9 (C-1), 172.1, 166.9 (C-2,3), 150.0 (C-4,8), 146.2 (C-12), 135.3, 135.1 (C-7,11), 134.7 (C-13), 130.4, 130.1 (C-6,6′,10,10′), 129.2 (C-14,16,18), 127.3 (C-15,17), 121.9 (C-5,5′,9,9′), 20.8(2 × CH_3_).

#### 4.2.2. 3-((2-(4,6-Diphenoxy-1,3,5-triazin-2-yl)hydrazono)methyl)phenol (2)

**Yield**: 89%; **m.p.**: 258–260°C; **R*_f_*** : 0.50 (chloroform : methanol; 9 : 1); **IR** (ATR, ῡ): cm^−1^ 3400–3000 (broad, O-H stretch.), 3232 (N-H stretch.), 3062 (C_sp2_—H stretch.), 1612 (C=N stretch.), 1571, 1544 (2 × C=C stretch.), 1368, 1191 (2 × C-O stretch.); **^1^H-NMR** (300 MHz, DMSO-d_6_): *δ* (ppm) 11.79 (1H, s, NH), 9.63 (1H, s, OH) 7.44 (1H, s, H-12), 7.44–7.42 (4H, m, H-6,6′,10,10′), 7.27–7.14 (8H, m, H-5,5′,9,9′,7,11,14,17), 7.00 (1H, d, *J* = 6.6 Hz, H-18), 6.80 (1H, d, *J* = 6.6 Hz, H-16); **^13^C-NMR** (75 MHz, DMSO-d_6_): *δ* (ppm) 172.8 (C-1), 171.9, 166.9 (C-2,3), 158.1 (C-15), 152.2 (C-4,8), 146.5 (C-12), 135.9 (C-15), 130.3 (C-17), 130.0, 129.8 (C-6,6′,10,10′) 126.1 (C-7,11), 122.2 (C-5,5′,9,9′), 119.0 (C-18), 117.7 (C-16), 112.9 (C-14).

#### 4.2.3. 3-((2-(4, 6-bis(4-Bromophenoxy)-1,3,5-triazinyl)hydrazono)methyl) phenol (3)

**Yield**: 71%; **m.p.**: 254°C; **R*_f_***: 0.60 (chloroform : methanol; 9 : 1); **IR** (ATR, ῡ): cm^−1^ 3500–2500 (broad, O-H stretch.), 3223 (N-H stretch.), 3124 (C_sp2_—H stretch.), 1607 (C=N stretch.), 1565, 1480 (2 × C=C stretch.), 1367, 1195 (2 × C-O stretch.); **^1^H-NMR** (300 MHz, DMSO-d_6_): *δ* (ppm) 11.81 (1H, s, NH), 9.61 (1H, s, OH), 8.08 (1H, s, H-12), 7.62 (4H, m, H-6,6′,10,10′), 7.27–7.19 (5H, m, H-5,5′,9,9′,17), 7.13 (1H, bs, H-14), 7.00 (1H, d, *J* = 7.2 Hz, H-18), 6.80 (1H, dd, *J =* 8.1, 2.4 Hz, H-16); **^13^C-NMR** (75 MHz, DMSO-d_6_): *δ* (ppm) 172.5 (C-1), 171.7, 166.8 (C-2,3), 158.1 (C-15), 151.4 (C-4,8), 135.8 (C-13), 132.8, 132.6 (C-6,6′,10,10′), 130.3 (C-17), 124.6 (C-5,5′,9,9′), 119.0 (C-18), 118.5, 118.3 (C-7,11), 117.8 (C-16), 113.0 (C-14).

#### 4.2.4. 4-((2-(4,6-bis(4-Chlorophenoxy)-1,3,5-triazin-2-yl)hydrazono)methyl-*N*,*N* dimethylaniline (4)

**Yield**: 93%; **m.p.**: 226–228°C; **R*_f_***: 0.80 (chloroform : methanol; 9 : 1); **IR** (ATR, ῡ): cm^−1^ 3243 (N-H stretch.), 3140 (C_sp2_—H stretch.), 2895 (C_sp3_—H stretch.), 1599 (C=N stretch.), 1572, 1483 (2 × C=C stretch.), 1366, 1193 (2 × C-O stretch.), 1084 (C-Cl); **^1^H-NMR** (300 MHz, DMSO-d_6_): *δ* (ppm) 11.56 (1H, s, NH), 8.03 (1H, s, H-12), 7.50–7.42 (6H, m, H-6,6′,10,10′,14,18), 7.31–7.24 (4H, m, H-5,5′,9,9′), 6.71 (2H, d, *J* = 8.7 Hz, H-15,17), 2.95 (6H, s, N(CH_3_)_2_); **^13^NMR**(75 MHz, DMSO-d_6_): (ppm) 172.6 (C-1), 171.6, 166.3 (C-2,3), 151.9 (C-16), 151.0 (C-4,8), 147.7 (C-12), 130.3, 130.1 (C-7,11), 129.9, 129.7 (C-6,6′,10,10′), 128.8 (C-14,18), 124.2 (C-5,5′,9,9′), 121.8 (C-13), 112.2 (C-15,14).

#### 4.2.5. 2-((*E*)-2-((*E*)-3-phenylallylidene)hydrazinyl)-4,6-bis(*p*-tolyloxy)-1,3,5-triazine (5)

**Yield**: 77%; **m.p.**: 225–227°C; **R*_f_***: 0.69 (chloroform : methanol; 9 : 1); **IR** (ATR, ῡ): cm^−1^ 3235 (N-H stretch.), 3124 (C_sp2_—H stretch.), 2916 (C_sp3_—H stretch.), 1625 (C = N stretch.), 1567, 1503 (2 × C = C stretch), 1366, 1193 (2 × C-O stretch); **^1^H-NMR** (300 MHz, DMSO-d_6_): *δ* (ppm) 11.67 (1H, s, NH), 7.98 (1H, dd, *J* = 6.6, 1.8 Hz, H-12), 7.59 (2H, d, *J* = 6.9 Hz, H-14,18), 7.40–7.30 (3H, m, H-15,16,17), 7.24–7.20 (4H, m, H-6,6′,10,10′), 7.13–7.08 (4H, m, H-5,5′,9,9′), 6.97 (2H, d, *J* = 6.6 Hz, H-X,Y), 2.32, 2.31 (6H, s, 2 × CH_3_); **^13^C-NMR** (75 MHz, DMSO-d_6_): *δ* (ppm); 172.9 (C-1), 172.1, 166.7 (C-2,3), 150.0 (C-4,8), 148.8 (C-12), 139.2 (C-13), 136.3 (C-X), 135.3, 135.1 (C-7,11), 130.4, 130.3 (C-6,6′,10,10′), 129.2 (C-14,15,17,18), 127.5 (C-16), 125.8 (C-Y), 121.87 (C-5,5′,9,9′), 20.8 (2 × CH_3_).

#### 4.2.6. 2,4-bis(4-Chlorophenoxy)-6-(2-((*E*)-3-phenylallylidene)hydrazinyl)-1,3,5-triazine (6)

**Yield**: 87%; **m.p**: 248–250°C; **R*_f_***: 0.73 (chloroform : methanol; 9 : 1); **IR** (ATR, ῡ): cm^−1^ 3240 (N-H stretch.), 3132 (C_sp2_—H stretch.), 1626 (C=N stretch.), 1574, 1485 (2 × C=C stretch.), 1366, 1209 (2 × C-O stretch.), 1085 (C-Cl); **^1^H-NMR** (300 MHz, DMSO-d_6_): *δ* (ppm) 11.76 (1H, s, NH), 7.99 (1H, dd, *J =* 5.1, 3.0 Hz, H-12), 7.61–7.47 (6H, m, H-6,6′,10,10′,H-15,17), 7.39–7.26 (7H, m, H-5,5′,9,9′,14,16,18), 6.99–6.97 (2H, m, H-19,20); **^13^C-NMR** (75 MHz, DMSO-d_6_): *δ* (ppm); 172.6 (C-1), 171.8, 166.6 (C-2,3), 151.0 (C-4,8), 149.2 (C-12), 139.5 (C-13), 136.3 (C-20), 130.4, 130.2 (C-7,11), 130.0, 129.8 (C-6,6′,10,10′), 129.3 (C-14,16,18), 127.6 (C-15,17), 125.7 (C-19), 124.2, 124.1 (C-5,5′,9,9′).

### 4.3. Crystallographic data collection and structural refinement

Single crystals of **1–6** were mounted on a thin glass fibre at room temperature and the reflection data were collected on a Bruker kappa APE XII CCD diffractometer equipped with graphite mono-chromated MoK*α* radiation (*λ* = 0.71073 Å). The data were also corrected for Lorentz and polarization effects. The structure was solved using SHELXS-97. Final refinement on F^2^ was carried out by full-matrix least-squares techniques using SHELXL-97 [50]. The crystal data of **1–6** (CCDC: 2155046-2155051) and refinement values are summarized in table 1.

## Data Availability

Data available as electronic supplementary material [51]. The crystallographic data can also be obtained from https://www.ccdc.cam.ac.uk/data_request/cif. CCDC: 2155046-2155051
